# Low levels of cerebrospinal fluid complement 3 and factor H predict faster cognitive decline in mild cognitive impairment

**DOI:** 10.1186/alzrt266

**Published:** 2014-06-23

**Authors:** Jon B Toledo, Ané Korff, Leslie M Shaw, John Q Trojanowski, Jing Zhang

**Affiliations:** 1 Pathology & Laboratory Medicine, Institute on Aging, Center for Neurodegenerative Disease Research, University of Pennsylvania School of Medicine, Philadelphia, PA, USA; 2 Department of Pathology, University of Washington School of Medicine, HMC Box 359635, 325 9th Avenue, Seattle, WA 98104, USA

## Abstract

**Introduction:**

Alzheimer’s disease (AD) is characterized by the deposition of tau and amyloid in the brain. Although the core cerebrospinal fluid (CSF) AD biomarkers amyloid β peptide 1–42 (Aβ_1–42_), total tau (t-tau) and phosphorylated tau 181 (p-tau_181_) show good diagnostic sensitivity and specificity, additional biomarkers that can aid in preclinical diagnosis or better track disease progression are needed. Activation of the complement system, a pivotal part of inflammation, occurs at very early stages in the AD brain. Therefore, CSF levels of complement proteins that could be linked to cognitive and structural changes in AD may have diagnostic and prognostic value.

**Methods:**

Using xMAP® technology based assays we measured complement 3 (C3) and factor H (FH) in the CSF of 110 controls (CN), 187 mild cognitive impairment (MCI) and 92 AD subjects of the AD Neuroimaging Initiative (ADNI) at baseline. All ADNI participants underwent clinical follow-up at 12 month intervals and MCI subjects had additional visits at 6 and 18 months. The association between CSF biomarkers and different outcome measures were analyzed using Cox proportional hazard models (conversion from MCI to AD), logistic regression models (classification of clinical groups) and mixed-effects models adjusted for age, gender, education, t-tau/Aβ_1–42_ and APOE ϵ4 presence (baseline and longitudinal association between biomarkers and cognitive scores).

**Results:**

Although no association was found between the complement proteins and clinical diagnosis or cognitive measures, lower levels of C3 (β = −0.12, p = 0.041) and FH (β = −0.075, p = 0.041) were associated with faster cognitive decline in MCI subjects as measured by the AD Assessment Scale-cognitive subscale (ADAS-Cog) test. Furthermore, lower FH levels were associated with larger lateral ventricular volume (p = 0.024), which is indicative of brain atrophy.

**Conclusions:**

Our study confirms a lack of suitability of CSF C3 and FH as diagnostic biomarkers of AD, but points to their modest potential as prognostic biomarkers and therapeutic targets in cognitively impaired patients.

## Introduction

Alzheimer’s disease (AD) affects an estimated 34 million people worldwide, a number that is predicted to triple by 2050 due to the aging population [1]. Despite intensive research and the identification of several promising drug candidates in preclinical studies, a neuroprotective treatment remains a major unmet need. Possible reasons for the failure of disease-modifying drug clinical trials include our inability to diagnose AD before substantial neuronal damage has occurred, as well as track disease progression and treatment response [2]. In this regard, sensitive and specific biomarkers are urgently needed.

The cerebrospinal fluid (CSF) biomarkers amyloid β peptide 1–42 (Aβ_1–42_), which correlates inversely with plaque pathology, total tau (t-tau), which is hypothesized to reflect neuroaxonal degeneration, and phosphorylated tau (p-tau_181_), which may correlate with tangle pathology, have recently been incorporated into the National Institute on Aging guidelines for AD diagnosis [3]. With regard to temporal dynamics, the decrease in CSF Aβ_1–42_ seems to be an early event, reaching a plateau before the onset of dementia and remaining relatively unchanged thereafter. The increase in CSF tau occurs after Aβ_1–42_ changes, but still in the preclinical stage of the disease and does not change appreciably over time in cognitively impaired subjects [4,5]. Therefore, although these core biomarkers show good sensitivity and specificity for the diagnosis of AD, additional biomarkers that can aid in the early diagnosis of dementia or can better track disease progression will improve the design and interpretation of clinical trials.

Neuroinflammation is generally recognized to be a major component of AD [6]. However, whether it is a cause, a contributing factor, or merely a consequence of neurodegeneration is unclear. Clinical and experimental evidence supports the involvement of inflammatory changes in the early stages of AD before the appearance of amyloid plaques [7], as well as in the progression of neurodegeneration [8]. If this is indeed the case, biomarkers that reflect the inflammatory process in AD hold promise for both early diagnosis and tracking of disease progression.

The complement system is a pivotal part of the immune system and inflammatory processes. Depending on the trigger, complement can be activated via the classical, alternative, or lectin pathways. All three pathways culminate in the formation of complement convertases, which results in the proteolytic cleavage of complement 3 (C3) and, later in the cascade, complement 5. The resulting active fragments act as proinflammatory and chemotactic anaphylatoxins, opsonins allowing phagocytosis, or anchors for the assembly of the membrane attack complex. The complement system is kept under tight control by soluble and membrane-bound regulators, including factor H (FH), which inhibits C3 convertases in the alternative pathway, and complement 1 inhibitor, which inhibits several proteases of the classical and lectin pathways [9]. The final outcome therefore depends on the balance between complement activation and inhibition, and dysregulation of this balance may contribute to neuroinflammation and disease [10]. As expected, complement activation has been shown to occur in the AD brain [9], even at very early stages of the disease [11]. Furthermore, genome-wide association studies have identified AD-associated variants at the complement receptor 1 gene [12,13], which correlate with a greater Aβ plaque burden and age-related cognitive decline [14]. Polymorphisms in the FH gene have also been linked to susceptibility to AD [15], although there are conflicting reports [16].

In a previous cross-sectional study, we found that CSF levels of C3 and FH were significantly increased in AD patients compared with controls (CN) and that the increase correlated significantly with lower Mini-Mental State Examination (MMSE) scores in AD patients. In the current study, we attempt to validate these observations in a large, independent cohort of well-characterized subjects. In addition, we extend the previous analysis by including patients diagnosed with mild cognitive impairment (MCI) and by analyzing additional clinical and neuroimaging data. Finally, we explore the prognostic potential of CSF C3 and FH levels by analyzing longitudinally collected clinical data.

## Methods

### Subjects

Data used in the current study were downloaded on 27 July 2013 from the Alzheimer’s Disease Neuroimaging Initiative (ADNI) database [17]. Ethics approval was obtained for each institution involved (see Acknowledgements and Additional file 1). The ADNI is conducted according to Good Clinical Practice guidelines, the Declaration of Helsinki, US 21CFR Part 50 – Protection of Human Subjects and Part 56 – Institutional Review Boards, and pursuant to state and federal Health Insurance Portability and Accountability Act regulations. Written informed consent is obtained from all subjects and/or authorized representatives and study partners before protocol-specific procedures are carried out. Institutional review boards were constituted according to applicable State and Federal requirements for each participating location. The protocols were submitted to the appropriate boards and their written unconditional approval obtained and submitted to Regulatory Affairs at the ADNI Coordinating Center prior to commencement of the study. The ADNI Coordinating Center supplied relevant data for investigators to submit to their hospital/university/independent institutional review boards for protocol review and approval. Verification of institutional review board unconditional approval of the protocol and the written informed consent statement with written information to be given to the participants and/or their authorized representatives and the study partners were transmitted and validated by the ADNI Coordinating Center in order to obtain approval for shipment of study supplies to study sites. The ADNI has previously been described extensively [18]. Criteria for the different diagnostic groups can be found in the ADNI procedures manual [19] (see also Additional file 2). In the current study, 389 ADNI 1 [20] subjects (110 CN, 187 MCI subjects and 92 AD subjects) had C3, FH, Aβ_1–42_, t-tau and p-tau_181_ measured in their CSF samples at baseline.

### Clinical assessment and cognitive profile

The same neuropsychological testing battery was applied to all subjects in the ADNI, with visits scheduled every 12 months, except for the MCI subjects who had additional visits at 6 and 18 months. Tests included the MMSE, the Alzheimer’s Disease Assessment Scale – cognitive subscale (ADAS-Cog), the clock drawing test, the Rey Auditory Verbal Learning Test, Digit Span forward and backward, category fluency, the trail-making test, the digit symbol substitution test, the Boston naming test and the logical memory test. Finally, we further characterized the cognitive profile of each subject using the summary composite executive function and memory measures developed by Gibbons and colleagues [21] and Crane and colleagues [22]. These measures summarize into a score factors from different tests that belong to the same cognitive domain, giving each test a specific loading and accounting for the difficulty of the different variations of the tests; for example, the different word lists available for the Rey Auditory Verbal Learning Test.

### Cerebrospinal fluid sample collection and analysis

CSF samples were obtained in the morning after an overnight fast at ADNI baseline visits. Lumbar puncture was performed with a 20-gauge or 24-gauge spinal needle as described previously [23] (for more details including sample handling and storage, see Additional file 2).

CSF C3 and FH levels were measured using an xMAP technology-based multiplex human neurodegenerative kit (HNDG1-36 K; Millipore, Billercia, MA, USA) according to the manufacturer’s overnight protocol with minor modifications. A detailed protocol can be found on the ADNI website [17]. Aβ_1–42_, t-tau and p-tau_181_ were measured using the multiplex xMAP Luminex platform (Luminex Corp, Austin, TX, USA) with an Innogenetics kit (INNO-BIA AlzBio3; Innogenetics, Ghent, Belgium) according to the manufacturer’s protocol [24]. CSF hemoglobin was measured using an ELISA kit from Bethyl Lab Inc. (Montgomery, TX, USA) according to the manufacturer’s instructions. Rules-based medicine (RBM; Austin, TX, USA) evaluated CSF samples using a multiplex Human DiscoveryMAP™ panel consisting of 159 analytes (including C3) on a Luminex 100 platform (Luminex Corp, Austin, TX, USA). More details on the methods used in the ADNI CSF Proteome study can be found on the ADNI website [25]. For details regarding each assay’s performance, see Additional file 2.

### Magnetic resonance imaging acquisition and processing

Acquisition of 1.5-T magnetic resonance imaging (MRI) data for the ADNI 1 subjects followed a previously described standardized protocol that included sagittal volumetric three-dimensional magnetization prepared rapid acquisition gradient recalled echo (MPRAGE), with variable resolution around the target of 1.2 mm isotropically. The scans had gone through certain correction methods such as gradwarp, B1 calibration, N3 correction and (in-house) skull-stripping (for details see [17,26]). The images were processed with a freely available pipeline [27] (for software see [28]). Briefly, images were segmented into three tissue types: grey matter, white matter and CSF. After high-dimensional image warping to an atlas, regional volumetric maps for grey matter, white matter and CSF were created – referred to herein as regional volumetric analysis of brain images, which are used for voxel-based analysis and group comparisons of regional tissue atrophy, as well as for constructing an index of AD brain morphology. We tested for differences in ventricular volume in our primary analysis and for associations with the different regions of interest in a secondary analysis.

### Statistical analysis

For the analyses included in the descriptive table (Table 1), one-way analyses of variance were used for quantitatively normally distributed variables and the data are presented as the mean (standard deviation). Kruskall–Wallis tests were used for quantitative non-normally distributed variables and the data are presented as the median (first quartile to third quartile). Chi-square tests were applied for qualitative variables and the data are presented as the percentage of counts. For further analyses, distributions of the variables and residuals were tested and power transformations applied if necessary. C3, FH and the C3/FH ratio were standardized to compare effect sizes across analytes.

**Table 1:** Alzheimer’s Disease Neuroimaging Initiative 1 subjects included in the study

	**Controls**	**MCI subjects**	**AD subjects**	***P *** **value**
Number of participants				
All	(*n* = 110)	(*n* = 187)	(*n* = 92)	
Hgb ≤ 1,500 ng/ml	(*n* = 96)	(*n* = 163)	(*n* = 83)	
Age at baseline (years)^a^				
All	75.4 (5.2)	74.0 (7.6)	74.4 (7.7)	0.24
Hgb ≤ 1,500 ng/ml	75.6 (5.0)	74.5 (7.6)	74.8 (7.9)	0.42
Gender (% female)^b^				
All	49.1	33.2	43.8	0.019
Hgb ≤ 1,500 ng/ml	46.9	33.7	47.0	0.046
Education (years)^a^				
All	15.7 (2.8)	15.8 (3.0)	15.0 (3.2)	0.15
Hgb ≤ 1,500 ng/ml	15.8 (2.8)	15.8 (3.0)	15.1 (3.2)	0.19
MMSE^a^				
All	29.1 (1.0)	27.0 (1.8)	23.6 (1.9)	<0.0001
Hgb ≤ 1,500 ng/ml	29.1 (1.0)	27.0 (1.8)	23.7 (1.9)	<0.0001
ADAS-Cog^a^				
All	9.7 (4.2)	18.6 (6.7)	28.5 (8.5)	<0.0001
Hgb ≤ 1,500 ng/ml	9.9 (4.1)	18.7 (6.2)	28.7 (7.7)	<0.0001
APOE ϵ4 presence (%)^b^				
All	24.5	54.0	70.7	<0.0001
Hgb ≤ 1,500 ng/ml	24.0	55.2	68.7	<0.0001
Hgb (ng/ml)^c^				
All	42.4 (0.0 to 418.8)	24.9 (0.0 to 477.7)	87.5 (0.0 to 338.4)	0.79
Hgb ≤ 1,500 ng/ml	19.6 (0.0 to 171.0)	0.64 (0.0 to 195.4)	42.9 (0.0 to 226.1)	0.40
Factor H (ng/ml)^c^				
All	1463.5 (1132.1 to 1954.1)	1478.9 (1177.6 to 2005.5)	1660.5 (1171.4 to 2274.1)	0.26
Hgb ≤ 1,500 ng/ml	1445.4 (1128.8 to 1877.1)	1452.0 (1176.3 to 1928.3)	1507.9 (1145.3 to 2053.2)	0.46
Complement 3 (ng/ml)^c^				
All	3331.4 (2595.5 to 4743.4)	3696.0 (2658.9 to 4905.2)	3894.6 (2567.6 to 5466.0)	0.40
Hgb ≤ 1,500 ng/ml	3331.4 (2644.1 to 4819.2)	3614.1 (2559.7 to 4597.7)	3690.3 (2522.3 to 5328.1)	0.73
Complement 3/factor H^c^				
All	2.40 (1.80 to 2.91)	2.42 (2.01 to 2.84)	2.20 (1.95 to 2.64)	0.31
Hgb ≤ 1,500 ng/ml	2.41 (1.86 to 2.95)	2.41 (1.98 to 2.82)	2.26 (1.95 to 2.78)	0.55
Aβ_1–42_ (pg/ml)^c^				
All	220.0 (159.0 to 253.0)	146.0 (128.0 to 202.0)	136.0 (120.8 to 160.5)	<0.0001
Hgb ≤ 1,500 ng/ml	220.0 (159.5 to 252.5)	146.0 (128.0 to 210.3)	137.0 (121.5 to 161.5)	<0.0001
Total tau (pg/ml)^c^				
All	61.0 (48.0 to 86.0)	87.0 (65.0 to 122.0)	110.5 (81.0 to 153.0)	<0.0001
Hgb ≤ 1,500 ng/ml	61.0 (48.0 to 79.5)	88.0 (65.0 to 126.0)	109.0 (77.0 to 143.0)	<0.0001
p-tau_181_ (pg/ml)^c^				
All	20.0 (16.0 to 29.0)	31.5 (21.0 to 45.0)	36.0 (29.0 to 49.0)	<0.0001
Hgb ≤ 1,500 ng/ml	19.0 (15.0 to 26.5)	31.0 (20.5 to 45.0)	35.0 (28.0 to 46.5)	<0.0001

Aβ_1–42_, amyloid β peptide 1–42; AD, Alzheimer’s disease; ADAS-Cog, Alzheimer’s disease Assessment Scale – cognitive subscale; APOE ϵ4, apolipoprotein E epsilon 4 allele; Hgb, hemoglobin; MCI, mild cognitive impairment; MMSE, Mini-Mental State Examination; p-tau_181_, tau phosphorylated at threonine 181. ^a^Mean (standard deviation), *P* value obtained using analysis of variance. ^b^*P* value obtained using the chi-square test. ^c^Median (first quartile to third quartile), *P* value obtained using the Kruskall–Wallis test.

Previous studies suggest that blood contamination of CSF can significantly affect CSF concentrations of certain proteins [29]. We therefore first tested whether hemoglobin levels were associated with complement biomarker levels in CSF, using a model that included gender, age, apolipoprotein E epsilon 4 allele presence (APOE ϵ4) and clinical diagnosis as covariates. CSF levels of FH (Figure S1a in Additional file 3) but not of C3 were significantly associated with hemoglobin. This association disappeared after exclusion of samples with hemoglobin levels > 1,500 ng/ml (14 CN, 24 MCI subjects, nine AD subjects) (Figure S1b in Additional file 3). All further analyses were therefore performed on the remaining 342 subjects. Exclusion of the 47 subjects with high hemoglobin levels did not significantly change the difference between diagnostic groups for any of the variables reported in Table 1.

Associations between CSF complement biomarkers and age, gender and APOE ϵ4 presence were tested in linear regression models. FH and C3, but not C3/FH, were associated with both age and gender. None of the CSF complement biomarkers showed a significant association with APOE ϵ4 presence (Table S1 in Additional file 4).

To test the classification accuracy of the analytes, we split the sample into a discovery set (67%) and a validation set (33%), stratifying by clinical diagnosis. To train a classifier and cross-validate the cutoff values in the discovery set, the subjects were further randomly split 10 times to form training (67%) versus test (33%) sets. The cutoff values of the model were selected in the discovery set using accuracy and the kappa index as performance metrics [30,31]. The obtained logistic regression model was then applied to the validation set and the sensitivity, specificity and the area under the curve of the receiver operating characteristic curve were obtained [32].

A Cox hazards model, with age, gender, t-tau/Aβ_1–42_ ratio, APOE ϵ4 presence and education as covariates was used to study the conversion of MCI to AD for different CSF biomarkers. Standardized values (mean = 0, standard deviation = 1) were used for the biomarker values in order to compare the effect size of the association.

We analyzed the cross-sectional and longitudinal association between CSF biomarkers and different outcome measures using mixed-effects models [33,34]. A mixed-effects model is an extension of a linear regression model that allows calculation of the mean trajectory of biomarker values for each group as well as the estimation of each patient’s trajectory. The mixed-effects model takes into account within-subject correlations from repeated measurements of biomarker values in the same subjects and for missing data points. Age, gender, education, APOE ϵ4 presence, t-tau/Aβ_1–42_ ratio and clinical diagnosis at baseline were included as fixed effects. We include an intercept, follow-up time and squared follow-up time in weeks as random effects. Our model specified the intercept and the regression coefficient for the follow-up time as random effects such that subjects have a unique intercept and slope characterizing their individual trajectories. An interaction between time and clinical diagnosis, between time and t-tau/Aβ_1–42_ ratio, and between time and the studied CSF biomarker was also included to assess whether these biomarkers were associated with the longitudinal change. A significant interaction between clinical diagnosis and time, for example, would indicate that the slope of change during follow-up is different in CN and MCI subjects. To plot the data we calculated the 25th, 50th and 75th percentile biomarker values for the MCI group and estimated the predicted changes based on the coefficients of the corresponding mixed-effects model (for the variables included in the model, median and mode values were used for quantitative and categorical predictors).

For the MRI analysis, a mixed-effects model was used, with a nested term inside subjects for the left and right sides for each region of interest. Age, gender and t-tau/Aβ_1–42_ ratio, clinical diagnosis and intracranial volume were included as fixed effects.

Statistical tests were two-sided and significance was set at *P* < 0.05. In the case of multiple comparisons, the Benjamini–Hochberg correction and the Holms method was applied when a large and lower number of comparisons, respectively, had been performed. Analyses were performed using R v. 3.0.1 [35].

## Results

A total of 389 ADNI 1 participants with CSF C3, FH, Aβ_1–42_, t-tau and p-tau_181_ as well as MRI data were included in the current study. Clinical and demographic characteristics of the studied subjects are summarized in Table 1. As expected, the clinical groups differed in gender, MMSE, ADAS-Cog, APOE ϵ4 presence and CSF Aβ_1–42_, t-tau and p-tau_181_. The data for CSF Aβ_1–42_, t-tau and p-tau_181_ in the AD subjects are in line with published cutoff values from autopsy confirmed cases using the AlzBio3 kit [23]. However, no differences in CSF C3 or FH levels or the C3/FH ratio were noted between the diagnostic groups. As discussed earlier, all subsequent analysis were performed on the 342 subjects whose CSF samples had hemoglobin levels ≤ 1,500 ng/ml. Exclusion of subjects with hemoglobin levels > 1,500 ng/ml (14 CN, 24 MCI subjects, nine AD subjects) did not seem to result in any selection bias (Table 1).

### Cross-sectional analysis

In linear regression analysis, a strong, positive correlation between C3 and FH (*r*_partial_ = 0.81, *P* < 0.0001) was present after adjusting for age and gender (Figure S2 in Additional file 3). In age, gender and APOE ϵ4 presence adjusted models, FH was significantly associated with t-tau but not with p-tau_181_ or Aβ_1–42_. C3 was not associated with Aβ_1–42_, t-tau or p-tau_181_ (Table S2 in Additional file 4).

Linear regression models adjusted for age, gender and APOE ϵ4 presence revealed no association of C3, FH or C3/FH with clinical diagnosis (Table S3 in Additional file 4). A lack of contribution from C3 and FH in classifying different clinical groups was further confirmed by the fact that the addition of C3, FH or C3/FH did not improve the performance of t-tau/Aβ_1–42_ in classifying AD subjects versus CN or MCI subjects versus CN (Table 2). The diagnostic utility of C3 and or FH was therefore not evaluated further.

**Table 2:** Classification accuracy of the cerebrospinal fluid biomarkers

	**AD subjects vs. controls**				**MCI subjects vs. controls**			
	**Sensitivity (%)**	**Specificity (%)**	**Kappa**	**AUC**	**Sensitivity (%)**	**Specificity (%)**	**Kappa**	**AUC**
Total tau/Aβ_1–42_	76.9	86.7	0.64	0.84	59.4	67.9	0.27	0.73
Total tau/Aβ_1–42_ C3	69.2	93.3	0.64	0.84	50.0	69.8	0.20	0.72
Total tau/Aβ_1–42_ FH	76.9	86.7	0.64	0.83	59.4	67.9	0.27	0.73
Total tau/Aβ_1–42_ C3/FH	76.9	86.7	0.64	0.84	56.3	66.0	0.22	0.71

Gender (male reference category) included as a covariate. Aβ_1–42_, amyloid β peptide 1–42; AD, Alzheimer’s disease; AUC, area under the curve; C3, complement 3; FH, factor H; MCI, mild cognitive impairment.

Finally, with regard to disease severity C3, FH and C3/FH did not associate with baseline ADAS-cog, MMSE, memory summary or executive function summary scores of AD (Table 3) or MCI (Table 4) subjects in mixed-effects models adjusted for age, gender, education, APOE ϵ4 presence and t-tau/Aβ_1–42_ ratio.

**Table 3:** Association of cerebrospinal fluid complement biomarkers with baseline and longitudinal changes (biomarker × time) in cognitive scores: Alzheimer’s disease subjects

**Model**	**Predictor**	**ADAS-Cog**		**MMSE**		**Memory**		**Executive**	
		**β**	***P *** **value**	**β**	***P *** **value**	**β**	***P *** **value**	**β**	***P *** **value**
C3	C3	−0.0095	1.0	−0.45	0.53	0.10	0.76	−0.061	1.0
	C3 × time	−0.0089	1.0	−0.009	1.0	0.085	0.18	0.12	0.092
FH	FH	−0.017	1.0	−0.14	0.54	0.083	0.59	−0.057	1.0
	FH × time	−0.0052	1.0	−0.0072	1.0	0.048	0.18	0.072	0.085
C3/FH	C3/FH	0.074	1.0	−1.25	0.51	−0.074	0.78	0.095	1.0
	C3/FH × time	0.014	1.0	−0.0075	1.0	0.092	0.42	0.081	0.57

Age, gender (male reference category), education, total tau/amyloid β peptide 1–42 ratio and apolipoprotein E epsilon 4 allele presence adjusted mixed-effects model. *P* values are corrected for multiple comparisons (Holms). ADAS-Cog, Alzheimer’s disease Assessment Scale – cognitive subscale; C3, complement 3; FH, factor H; MMSE, Mini-Mental State Examination.

**Table 4:** Association of cerebrospinal fluid complement biomarkers with baseline and longitudinal changes (biomarker × time) in cognitive scores: mild cognitive impairment subjects

**Model**	**Predictor**	**ADAS-Cog**		**MMSE**		**Memory**		**Executive function**	
		**β**	***P *** **value**	**β**	***P *** **value**	**β**	***P *** **value**	**β**	***P *** **value**
C3	C3	−0.061	1.0	−0.062	1.0	−0.050	1.0	0.20	0.18
	C3 × time	−0.12	0.041	0.40	0.16	0.067	0.051	0.028	0.70
FH	FH	−0.077	1.0	−0.17	1.0	−0.014	1.0	0.077	0.29
	FH × time	−0.075	0.041	0.29	0.15	0.037	0.089	0.26	0.51
C3/FH	C3/FH	0.076	1.0	0.49	1.0	−0.13	1.0	0.41	0.18
	C3/FH × time	−0.18	0.057	0.34	0.44	0.10	0.089	−0.0013	0.98

Age, gender (male reference category), education, total tau/amyloid β peptide 1–42 ratio and apolipoprotein E epsilon 4 allele presence adjusted mixed-effects model. *P* values are corrected for multiple comparisons (Holms). ADAS-Cog, Alzheimer’s disease Assessment Scale – cognitive subscale; C3, complement 3; FH, factor H; MMSE, Mini-Mental State Examination.

### Longitudinal analysis

Of the 160 MCI subjects included in the analysis, 79 converted to AD with a median follow-up time of 158 weeks. A Cox hazards model with age at baseline (hazard ratio = 1.01, *P* = 0.59), gender (hazard ratio = 1.05, *P* = 0.84), t-tau/Aβ_1–42_ ratio (hazard ratio = 1.49, *P* = 0.002), APOE ϵ4 presence (hazard ratio = 1.13, *P* = 0.63) and education (hazard ratio = 1.02, *P* = 0.70) as covariates was used to test the association of the CSF complement biomarkers with conversion of MCI to AD. A weak association between lower levels of C3 (hazard ratio = 0.62, *P*_unadj_ = 0.046) and increased conversion was lost after adjustment for multiple comparisons (*P*_adj_ = 0.14).

**Figure 1:**
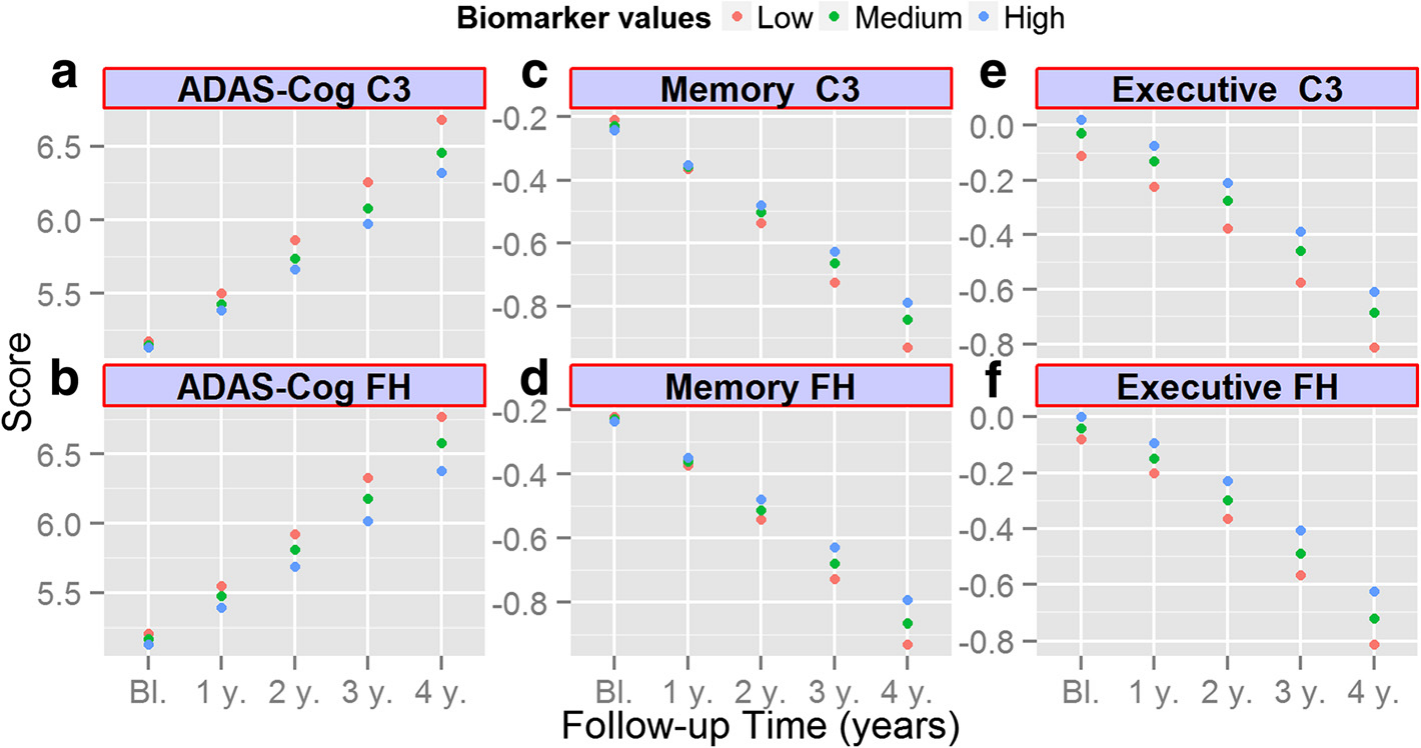
**Association of cerebrospinal fluid complement 3 and factor H levels with longitudinal cognitive changes in mild cognitive impairment subjects. (a)**, **(b)** Alzheimer’s disease Assessment Scale – cognitive subscale (ADAS-Cog), **(c)**, **(d)** summary memory scores and **(e)**, **(f)** summary executive function scores are represented on the *y* axis, with follow-up time on the *x* axis. Although the variables examined here were treated as quantitative in the analysis, the graphs represent the different tertiles for ease of visual representation. C3, complement 3; FH, factor H.

Longitudinal ADAS-Cog, MMSE, memory summary and executive function summary scores were next analyzed against baseline CSF C3 and FH levels. Follow-up of ADNI 1 patients with a baseline diagnosis of AD was discontinued at an earlier time point than baseline MCI patients (mean follow-up was 98.3 weeks for the AD group and 184 weeks for the MCI group). Owing to this imbalance in the number of visits, we analyzed the AD subjects and the MCI subjects separately. All of the following analyses were adjusted for age, gender, APOE ϵ4 presence, education and t-tau/Aβ_1–42_ ratio. In the analysis of the AD group, none of the complement biomarkers were associated with changes in ADAS-Cog, MMSE, memory summary, or executive function summary scores during follow-up (Table 3). In the MCI subjects, lower levels of both C3 and FH were associated with an increase (more severe cognitive impairment) in ADAS-Cog scores during follow-up (biomarker × time interaction, Table 4; Figure 1a,b). The C3/FH ratio showed no association with longitudinal ADAS-Cog score changes. Finally, none of the CSF complement biomarkers showed a significant association with MMSE, memory summary, or executive function summary scores, although there was a trend for an association of lower C3 levels with a decline in memory summary score during follow-up (biomarker × time interaction, Table 4; Figure 1c,d,e,f).

### Correlations of complement 3 and factor H with other ADNI variables

A subset (*n* = 256) of the ADNI 1 subjects included in the study also had RBM CSF data available. The RMB CSF panel included C3, but not FH. We explored the association between our CSF C3 measurements and the RBM C3 levels and found a strong correlation between the two (*r* = 0.79, *P* < 0.0001), indicating that the two immunoassays performed similarly. As expected, when we repeated the diagnostic and prognostic analyses described above using C3 levels obtained by the RBM assay, very similar, if not identical, results were found, especially with regard to the association between lower levels of C3 and faster cognitive decline of MCI subjects.

Since brain atrophy detected by MRI is associated with AD severity and correlates closely with changes in cognitive performance [36], we also studied the association between the CSF complement biomarkers and MRI volumes at baseline in a model adjusted for age, gender, clinical diagnosis, t-tau/Aβ_1–42_ ratio and intracranial volume. There were no associations between C3 levels or the C3/FH ratio and MRI volumes. However, although no association with CSF FH levels was noted when different region of interest were analyzed independently (Table S4 in Additional file 4), low FH values were clearly associated with increased lateral ventricular volume in the multiple comparison-adjusted analyses (*P*_adj_ = 0.024), consistent with the argument that low CSF FH level is associated with greater brain atrophy.

## Discussion

In this study, we explored the diagnostic and prognostic value of CSF C3 and FH levels in AD and MCI. In cross-sectional analysis, there were no significant differences in either biomarker or in their ratio between diagnostic groups, and nor were there any correlations with disease severity in AD or MCI subjects as measured by the MMSE, ADAS-Cog, memory summary score or executive function summary score. In the longitudinal analysis of MCI patients, however, low levels of both C3 and FH were modestly associated with an increase in ADAS-Cog scores (more severe cognitive impairment) and validation in an independent and longitudinal cohort is needed. Additionally, there was a significant association of low CSF FH levels with increased lateral ventricular volume, which is indicative of brain atrophy and has been shown to correlate strongly with changes in cognitive tests [37]. Strengths of the current study include the use of a large cohort of subjects that underwent detailed clinical and neuropsychological testing and longitudinal follow-up, the availability of APOE ϵ4 genotype data, hemoglobin measurements to control for blood contamination of CSF, as well as the use of RBM CSF C3 data to corroborate our own measurements and results.

Previous studies have generally reported elevated levels of complement components in AD CSF, although results are inconsistent. For example, in a recent study [38] using a commercially available ELISA kit, CSF C3 levels were increased in AD patients and CN subjects compared with stable MCI subjects, but there was no significant difference between AD patients and CN subjects, or between the MCI-to-AD group and any of the other groups. Consistent with the current finding, receiver operating characteristic analysis revealed no diagnostic utility for CSF C3. On the other hand, in a study using the RBM Human DiscoveryMAP™ panel on a Luminex 100 platform, CSF C3 levels were increased in autopsy-confirmed AD cases compared with normal controls. Furthermore, there was a significant correlation between CSF C3 levels and MMSE scores in AD subjects, but not in MCI subjects [39]. In contrast, a study using two-dimensional electrophoresis found no significant difference in the average percent volume for C3b or FH in CSF samples from AD compared with normal controls [40]. In line with the RBM study discussed above, our own earlier study found increased CSF C3 and FH levels in AD patients compared with CN subjects, as well as significant negative correlations between the two complement biomarkers and MMSE scores [41]. The failure to validate our own earlier findings in the current study may be related to differences between the two cohorts, including highly selected subjects in the ADNI versus the community-based cohort in our previous study, gender distribution and mean age, as well as the number of AD patients included in the analysis (38 in the previous study vs. 83 in the current study). One should also point out that although our original study found significant differences in CSF C3 and FH levels between diagnostic groups, receiver operating characteristic analysis showed that neither biomarker had acceptable sensitivity or specificity (>60%) for classifying CN versus AD subjects. In summary, we conclude that these studies are in agreement regarding a lack of suitability of CSF C3 and FH as diagnostic biomarkers of AD.

A limitation of our investigation is the potential confounding effect of pharmacotherapy, because subjects were not drug naïve at the time of CSF collection, although the use of many central nervous system-active drugs such as antidepressants or neuroleptics with anti-cholinergic properties, narcotic analgesics and anti-Parkinsonian medications were excluded. The other limitation relates to the fact that the data are correlational without clear mechanistic interpretation. That said, we wish to put forward two hypotheses for discussion. First, our finding of low levels of CSF C3 and FH in MCI patients with accelerated cognitive decline may reflect increased deposition of these complement biomarkers in senile plaques. Decreased Aβ_1–42_ in AD CSF is hypothesized to be the result of trapping the peptide in plaques, and C3 and FH have both been shown to be present in Aβ plaques [11,42,43]. Trapping of C3 and FH in plaques may therefore lead to a decrease in the CSF levels of these proteins. However, we did not find a correlation between CSF Aβ_1–42_ and C3 or FH, suggesting that the observed decrease in complement biomarkers cannot be readily explained by such a simplistic model. An alternative hypothesis could therefore be that the low CSF levels of C3 and FH in faster progressors may reflect accelerated dysregulation of the complement system in the brain. To this end, many studies have indicated potential involvement of complement system in AD pathogenesis, including observations that: Aβ fibrils activate both the classical and alternative complement pathways *in vitro*[44-46]; inhibition of C3 in a mouse model of AD resulted in accelerated and increased Aβ plaque deposition, as well as neurodegeneration [47-49]; and AD mice lacking C1q (part of the complex triggering activation of the classical pathway) had decreased levels of activated glia in proximity to plaques, as well as reduced neuronal injury [50] consistent with a detrimental role for complement activation in this model. Indeed, based on these and other studies, a hypothesis has been suggested that classical complement activation is detrimental in neurodegeneration, whereas alternative complement activation is beneficial up to a certain threshold or depending on the complement receptor *CR1* genotype [51]. Thus, if CSF levels of C3 and FH mirror their levels in the brain, our finding of a decreased total C3 level may indicate increased cleavage of C3 to generate more of its active fragments at the expense of the holoprotein. This increased activation of C3 might be due to increased activation of the classical, lectin and/or alternative pathways, as C3 is a joining point for all three. Decreased levels of FH will also lead to increased cleavage of C3 via the alternative pathway, because FH regulates this pathway at the C3 convertase level [9]. The strong correlation observed between CSF C3 and FH in the current study supports this hypothesis. The negative finding with regard to the longitudinal analysis in AD patients could be secondary to the much shorter followup in these patients and a smaller sample size, which results in less statistical power.

## Conclusions

In summary, our data suggest that CSF C3 and FH levels are prognostic biomarkers of accelerated cognitive decline in MCI, although validation in an independent cohort is needed. Additionally, studies with repeated CSF measurements will shed more light on the utility of CSF C3 and FH levels as AD progression biomarkers. Finally, results obtained in this study should encourage further investigations exploring the mechanisms underlying complement activation, both the classical and alternative cascades, in AD development and progression.

## Abbreviations

Aβ_1–42_: amyloid β peptide 1–42; AD: Alzheimer’s disease; ADAS-Cog: Alzheimer’s Disease Assessment Scale – cognitive subscale; ADNI: Alzheimer’s Disease Neuroimaging Initiative; APOE ϵ4: apolipoprotein E epsilon 4 allele; C3: complement 3; CN: controls; CSF: cerebrospinal fluid; FH: factor H; MCI: mild cognitive impairment; MMSE: Mini-Mental State Examination; MRI: magnetic resonance imaging; p-tau_181_: tau phosphorylated at threonine 181; RBM: rules-based medicine; t-tau: total tau.

## Competing interests

The authors declare that they have no competing interests.

## Authors’ contributions

JBT, AK, LMS, JQT and JZ made substantial contributions to the conception and design of the study and were involved in drafting the manuscript and revising it critically for important intellectual content. AK performed the xMAP assays. JBT, AK and JZ had full access to the entire dataset. LMS and JQT contributed to acquisition and storage of the samples. JBT and AK undertook the statistical analyses. All authors read and approved the final manuscript.

## Supplementary Material

Additional file 1 is an acknowledgement list for ADNI publications: ADNI infrastructure and site investigators. Click here for file

Additional file 2 is the supplemental methods, including subjects, recruitment criteria, CSF sample collection and handling, CSF immunoassay performance and references. Click here for file

Additional file 3 is Supplemental Figures S1 and S2 showing associations between CSF FH and hemoglobin, and CSF C3 and FH. Click here for file

Additional file 4 is Supplemental Tables S1 to S4 presenting data showing CSF C3 and FH biomarker associations. Click here for file
