# Supplementary material for: Low levels of cerebrospinal fluid complement 3 and factor H predict faster cognitive decline in mild cognitive impairment
Source: Alzheimers Res Ther. 2014 Jun 23;6(3):36. doi: 10.1186/alzrt266 (PMC4255518; doi:10.1186/alzrt266)
Supplement: Additional file 2 — is the supplemental methods, including subjects, recruitment criteria, CSF sample collection and handling, CSF immunoassay performance and references. [file alzrt266-S2.pdf]

Supplemental methods: Low levels of cerebrospinal fluid complement 3 and factor H predict faster cognitive decline in mild cognitive impairment

Jon B. Toledo, Ané Korff, Leslie M. Shaw, John Q. Trojanowski and Jing Zhang for the Alzheimer's Disease Neuroimaging Initiative

1. Subjects

ADNI was launched in 2004 by the NIA, the National Institute of Biomedical Imaging and Bioengineering (NIBIB), the Food and Drug Administration, private pharmaceutical companies and non-profit organizations as a public- private partnership. The primary goal of ADNI has been to test whether serial magnetic resonance imaging (MRI) [1, 2], positron emission tomography (PET) [3], CSF tau and Aβ, as well as other biological markers [4] and clinical and neuropsychological assessment [5] can be combined to measure the progression of MCI and early AD, as well as the conversion of NC subjects to MCI or AD. The initial ADNI study (ADNI 1) has been renewed (ADNI 2) to continue to 2016. Determination of sensitive and specific markers of very early AD progression is intended to aid researchers and clinicians to develop new treatments and monitor their effectiveness, as well as lessen the time and cost of clinical trials. The Principal Investigator of this initiative is Michael W. Weiner, MD, VA Medical Center and University of California – San Francisco. ADNI is the result of efforts of many co-investigators from a broad range of academic institutions and private corporations.

2. Recruitment inclusion and exclusion criteria for ADNI 1

Inclusion criteria were as follows: 1) Hachinski Ischemic Score ≤4; 2) Permitted medications stable for 4 weeks prior to screening; 3) Geriatric Depression Scale score < 6; 4) visual and auditory acuity adequate for neuropsychological testing; good general health with no diseases precluding enrollment; 5) 6 grades of education or work history equivalent; 6) Ability to speak English or Spanish fluently; 7) A study partner with 10 hours per week of contact either in person or on the telephone and who could accompany the participant to the clinical visits.

Groups were age matched. CN subjects could not have any significant cognitive impairment or impaired activities of daily living. AD had mild AD and had to meet the National Institute of Neurological and Communicative Disorders and Stroke–Alzheimer's Disease and Related Disorders Association criteria for probable AD [6], whereas MCI subjects should not meet this criteria and have largely intact general cognition and functional performance.

3. CSF sample collection and handling in ADNI 1

CSF in the ADNI was collected into polypropylene collection tubes provided to each site, then transferred into polypropylene transfer tubes, frozen on dry ice within 1 hour after collection and shipped overnight on dry ice to the ADNI Biomarker Core laboratory at the Perelman School of Medicine of the University of Pennsylvania. Aliquots (0.5 ml) were prepared from these samples after thawing (1 hour) at room temperature and gentle mixing. The aliquots were stored in bar code–labeled polypropylene vials at 80°C.

4. CSF immunoassay performance

Intra-assay variability for the human neurodegenerative kit (HNDG1-36K; Millipore, Billercia, MA) is 3 % and inter-assay variability is 7-8 %. The lower limit of quantification (LLOQ) is 0.05 ng/ml for C3 and 0.223 ng/ml for FH, and the accuracy based on spike recovery is 92 % for C3 and 98 % for FH.

Intra-assay variability for the Innogenetics AlzBio3 kit is < 4 % and inter-assay variability is < 10 %. LLOQ is 30-40 pg/ml for t-tau, 20-50 pg/ml for $A\beta_{1-42}$ and 8-10 pg/ml for p-tau_{181} [7, 8]. Further details regarding the qualification of the analytical and clinical performance of the AlzBio3 kit are described in Shaw et al [4, 9].

The CSF hemoglobin ELISA assay range is 6.25 – 400 ng/ml. Samples were run in duplicate, with CVs generally being < 10 %. Any samples with CV ≥ 20 % were rerun.

MyriadRBM attempted to validate each of the analytes on the 159 analyte panel up to clinical laboratory improvement amendment (CLIA) standards, but the assays themselves are not CLIA approved. Each analyte has an individual standard curve with between 6-8 reference standards. Each plate is run with 3

levels of QCs (low, medium and high) for each analyte. A total of 16 of the CSF samples were retested using a separate never before thawed replicate aliquot on the fifth of the five 96 well plates to provide blinded test/re-test quality control data. Assays are qualified based on least detectable dose (LDD - see below), precision, cross-reactivity, dilution linearity, spike recovery (assessment of accuracy), and test/re-test performance.

References

1. Jack CR, Jr., Bernstein MA, Fox NC, Thompson P, Alexander G, Harvey D, Borowski B, Britson PJ, J LW, Ward C, et al: **The Alzheimer's Disease Neuroimaging Initiative (ADNI): MRI methods.** *Journal of magnetic resonance imaging : JMRI* 2008, **27:**685-691.
2. Weiner MW, Veitch DP, Aisen PS, Beckett LA, Cairns NJ, Green RC, Harvey D, Jack CR, Jagust W, Liu E, et al: **The Alzheimer's Disease Neuroimaging Initiative: a review of papers published since its inception.** *Alzheimer's & dementia : the journal of the Alzheimer's Association* 2012, **8:**S1-68.
3. Jagust WJ, Bandy D, Chen K, Foster NL, Landau SM, Mathis CA, Price JC, Reiman EM, Skovronsky D, Koeppe RA: **The Alzheimer's Disease Neuroimaging Initiative positron emission tomography core.** *Alzheimer's & dementia : the journal of the Alzheimer's Association* 2010, **6:**221-229.
4. Shaw LM, Vanderstichele H, Knapik-Czajka M, Clark CM, Aisen PS, Petersen RC, Blennow K, Soares H, Simon A, Lewczuk P, et al: **Cerebrospinal fluid biomarker signature in Alzheimer's disease neuroimaging initiative subjects.** *Annals of neurology* 2009, **65:**403-413.
5. Petersen RC, Aisen PS, Beckett LA, Donohue MC, Gamst AC, Harvey DJ, Jack CR, Jr., Jagust WJ, Shaw LM, Toga AW, et al: **Alzheimer's Disease Neuroimaging Initiative (ADNI): clinical characterization.** *Neurology* 2010, **74:**201-209.
6. McKhann GM, Knopman DS, Chertkow H, Hyman BT, Jack CR, Jr., Kawas CH, Klunk WE, Koroshetz WJ, Manly JJ, Mayeux R, et al: **The diagnosis of dementia due to Alzheimer's disease: recommendations from the National Institute on Aging-Alzheimer's Association workgroups on diagnostic guidelines for Alzheimer's disease.** *Alzheimer's & dementia : the journal of the Alzheimer's Association* 2011, **7:**263-269.
7. Olsson A, Vanderstichele H, Andreasen N, De Meyer G, Wallin A, Holmberg B, Rosengren L, Vanmechelen E, Blennow K: **Simultaneous measurement of beta-amyloid(1-42), total tau, and phosphorylated tau (Thr181) in cerebrospinal fluid by the xMAP technology.** *Clinical chemistry* 2005, **51:**336-345.
8. Vanderstichele H, DeMeyer L, DeRoo K, Van Remoortere S, Mortier V, Olsson A, Vandecasteele L, Kostanjevecki V, Blennow K, Vanmechelen E: **Standardized multiparameter quantification of biomarkers for Alzheimer’s disease in cerebrospinal fluid.** In *New trends in Alzheimer and Parkinson related disorders.* Edited by Fisher A, I. H, Memo M, Stocchi F. Bologna, Italy: Medimond S.r.l.; 2005: 183–189.[ADPD (Series Editor)
9. Shaw LM, Vanderstichele H, Knapik-Czajka M, Figurski M, Coart E, Blennow K, Soares H, Simon AJ, Lewczuk P, Dean RA, et al: **Qualification of the analytical and clinical performance of CSF biomarker analyses in ADNI.** *Acta neuropathologica* 2011, **121:**597-609.
