# Supplementary material for: Low levels of cerebrospinal fluid complement 3 and factor H predict faster cognitive decline in mild cognitive impairment
Source: Alzheimers Res Ther. 2014 Jun 23;6(3):36. doi: 10.1186/alzrt266 (PMC4255518; doi:10.1186/alzrt266)

Supplemental figures: Low levels of cerebrospinal fluid complement 3 and factor H predict faster cognitive decline in mild cognitive impairment

Jon B. Toledo, Ané Korff, Leslie M. Shaw, John Q. Trojanowski and Jing Zhang for the Alzheimer's Disease Neuroimaging Initiative

Supplemental figure 1. Association of FH with hemoglobin levels in CSF. (a) All samples (b) Higher magnification graph showing samples with hemoglobin ≤1500 ng/ml.

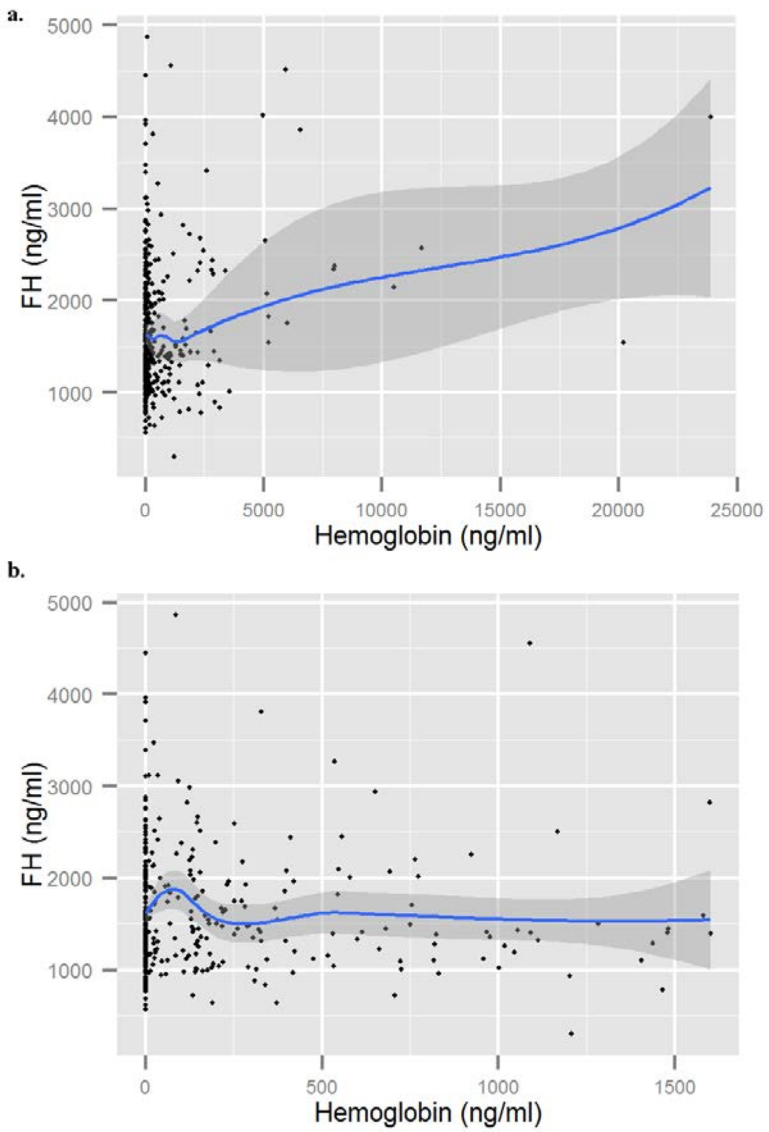

Supplemental figure 2. Association between CSF C3 and FH levels.

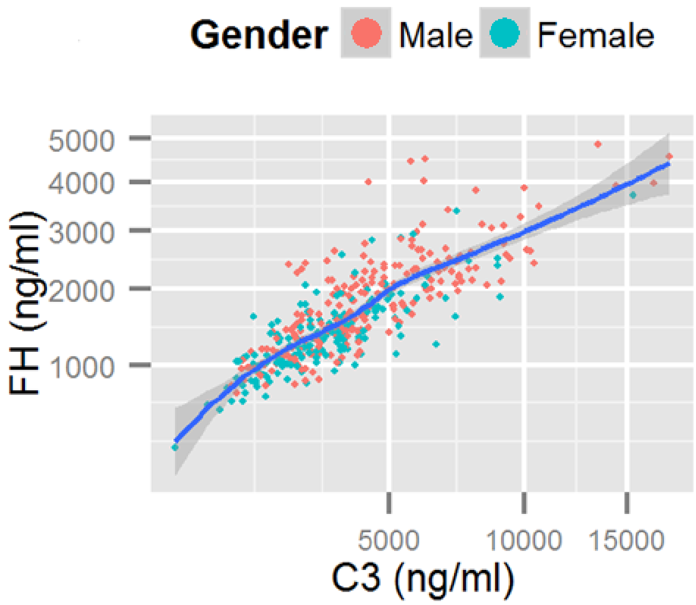

Supplement: Additional file 3 — is Supplemental Figures S1 and S2 showing associations between CSF FH and hemoglobin, and CSF C3 and FH. [file alzrt266-S3.pdf]
