# Supplementary material for: Low levels of cerebrospinal fluid complement 3 and factor H predict faster cognitive decline in mild cognitive impairment
Source: Alzheimers Res Ther. 2014 Jun 23;6(3):36. doi: 10.1186/alzrt266 (PMC4255518; doi:10.1186/alzrt266)
Supplement: Additional file 4 — is Supplemental Tables S1 to S4 presenting data showing CSF C3 and FH biomarker associations. [file alzrt266-S4.pdf]

Supplemental tables: Low levels of cerebrospinal fluid complement 3 and factor H predict faster cognitive decline in mild cognitive impairment

Jon B. Toledo, Ané Korff, Leslie M. Shaw, John Q. Trojanowski and Jing Zhang for the Alzheimer's Disease Neuroimaging Initiative

Supplemental table 1. Association of CSF complement biomarkers with age, gender and APOE ε4 presence.

	Age		Gender (Female)		APOE ε4 presence	
	β	p-value	β	p-value	β	p-value
C3	0.011	0.0047	-0.24	<0.0001	-0.10	0.075
FH	0.025	0.0001	-0.53	<0.0001	-0.086	0.36
C3/FH	-0.0003	0.91	-0.012	0.70	-0.064	0.0501

Age, gender (male reference category), clinical diagnosis and APOE ε4 presence adjusted linear regression model. P-values are not adjusted for multiple comparisons.

Supplemental table 2. CSF biomarker associations.

	$A\beta_{1-42}$		T-Tau		$P\text{-}Tau_{181}$	
	β	p-value	β	p-value	β	p-value
C3	0.20	p=0.09	0.012	p=1.0	-0.015	p=1.0
FH	0.27	p=0.095	0.26	p=0.010	0.18	p=0.095

Age, gender (male reference category) and APOE ε4 presence adjusted linear regression model. P-values adjusted for multiple comparisons (Holms).

Supplemental table 3. Association between CSF complement biomarkers and clinical diagnosis.

	MCI		AD	
	β	p-value	β	p-value
C3	0.016	1.0	0.11	0.32
FH	-0.021	1.0	-0.086	0.23
C3/FH	0.027	1.0	0.013	0.77

Age, gender (male reference category) and APOE ε4 presence adjusted linear regression model using CN group as reference group. P-value adjusted for multiple comparisons (Holms).

Supplemental table 4. Association between C3, FH and C3/FH ratio and cortical grey matter.

ROI	C3	FH	C3/FH
Inferior frontal cortex	0.94	0.82	0.82
Insular cortex	0.82	0.93	0.75
Lateral frontal cortex	0.94	0.93	0.97
Frontal medial cortex	0.94	0.92	0.93
Opercular cortex	0.82	0.92	0.82
Cingulate cgyrus	0.68	0.68	0.93
Medial temporal cortex	0.89	0.82	0.93
Occipital inferior cortex	0.97	0.82	0.79
Occipital lateral cortex	0.68	0.82	0.68
Occipital medial cortex	0.93	0.97	0.89
Lateral parietal cortex	0.92	0.96	0.82
Lateral medial cortex	0.68	0.83	0.68
Inferior temporal cortex	0.68	0.75	0.68
Lateral temporal cortex	0.68	0.75	0.68
Superior temporal cortex	0.68	0.75	0.82

Age, gender (male reference category), education, clinical diagnosis, APOE ε4 presence and t-tau/A$\beta_{1\text{-}42}$ ratio adjusted linear regression model. P-values adjusted for multiple comparisons. ROI = region of interest.
